# Unveiling Equine Abortion Pathogens: A One Health Perspective on Prevalence and Resistance in Northwest China

**DOI:** 10.3390/pathogens14121275

**Published:** 2025-12-11

**Authors:** Wei Gao, Mengyao Liu, Kastai Nurdaly, Duojie Caidan, Yunlong Sun, Jingang Duan, Jiangshan Zhao, Xiaowei Gong, Jizhang Zhou, Yong Zhang, Qiwei Chen

**Affiliations:** 1 College of Veterinary Medicine, Gansu Agricultural University, Lanzhou 730070, China; 17834315379@163.com; 2 State Key Laboratory of Animal Disease Control and Prevention, Lanzhou Veterinary Research Institute, Chinese Academy of Agricultural Sciences, College of Animal Medicine and Biosafety, Lanzhou University, Lanzhou 730030, China; lmy11292025@163.com (M.L.); haster8182@163.com (K.N.); doran82@163.com (D.C.); 13997415725@163.com (Y.S.); djg320142@163.com (J.D.); gongxiaowei@caas.cn (X.G.); zhoujizhang@caas.cn (J.Z.); 3 Xinjiang Uygur Autonomous Region Center for Disease Control and Prevention, Urumqi 830063, China; zjscdc@163.com

**Keywords:** equine abortion, *Salmonella abortus equi*, *Coxiella burnetii*, antimicrobial resistance genes, northwest China

## Abstract

Equine bacterial abortion presents substantial economic and One Health challenges; however, comprehensive epidemiological data from China are limited. This study sought to ascertain the overall prevalence of key pathogens—namely, *Chlamydia* spp., *Coxiella burnetii*, *Salmonella abortus equi*, and *Brucella* spp.—in equine populations in northwestern China. In this study, we aimed to further elucidate the characteristics of co-infections, profile antimicrobial resistance genes, and identify associated risk factors. Conducted as a cross-sectional analysis across four provinces, we collected 508 blood samples and 24 abortion tissue samples from 15 farms. Pathogen detection was performed using ELISA and real-time PCR, complemented by a targeted PCR panel screening for 29 AMR genes. The highest prevalence was observed for *S. abortus equi* (serology: 35.03%; molecular: 23.03%), followed by *C. burnetii* (28.94%; 15.35%) and *Chlamydia* spp. (18.90%; 14.17%). No PCR-confirmed cases of *Brucella* spp. were detected, despite low-level seropositivity. Notably, donkeys and horses aged 5–10 years exhibited higher positivity rates, and co-infections were common, particularly *S. abortus equi* + *C. burnetii* (*n* = 44). Among the 196 PCR-positive samples, extended-spectrum beta-lactamase (ESBL) genes were predominant, with *CTX-M* (n = 158) and *TEM-1* (n = 106) being the most prevalent. Additionally, we identified a high prevalence of genes conferring resistance to fluoroquinolones *(qnrA/B*), tetracyclines (*tetM*), macrolides (*ermA/B/C*), and sulfonamides *(sul1*), along with sporadic occurrences of carbapenemase genes. This study presents the inaugural comprehensive analysis of pathogen prevalence and associated antimicrobial resistance (AMR) gene carriage in equine abortion cases in northwest China. The findings highlight the imperative for integrated serological and molecular surveillance, revealing a significant discrepancy between empirical therapeutic approaches and the prevalent resistance genotypes. Consequently, this research lays the groundwork for evidence-based biosecurity measures and antimicrobial stewardship within a One Health framework.

## 1. Introduction

Equine abortion is one of the most devastating reproductive disorders faced by the global horse industry, posing an ongoing threat to the economy, genetic resource preservation, and even the survival of specific breeds. The severity of this issue was brutally confirmed in the 1980s [1], when a global equine abortion crisis led to the miscarriage of tens of thousands of pregnant mares, causing billions of dollars in economic losses. This event remains a cautionary reminder for the industry to this day. In China, horses, as important agricultural assets and cultural symbols, are experiencing a resurgence due to the expansion of emerging markets such as horse racing, equestrian sports, and biological products (such as equine serum). This trend is particularly evident in the northwestern provinces of Xinjiang, Qinghai, and Gansu, where the combination of traditional livestock farming and growing market demand has driven significant increases in horse population and breeding activities. However, accompanying this growth is the increasing frequency of abortion outbreaks, which have become a bottleneck restricting the healthy development of the local horse industry. The economic losses are substantial, but the resulting genetic resource loss, farmer discouragement, and the cascading impact on related industries make addressing equine abortion an urgent priority.

The causes of abortion in pregnant mares are complex, involving management, nutrition, genetics, and infectious factors. Among these, infectious pathogens, particularly bacterial pathogens, have become the focus of prevention and control due to their potential for rapid transmission and the risk of triggering an “abortion storm.” A wide variety of pathogens are known to cause equine abortion, including viral pathogens like *Equine Herpesvirus 1 (EHV-1)* [2,3] and *Equine Viral Arteritis (EVA)* [4]; parasitic pathogens such as *Equine Trypanosomes* [5]; and numerous bacterial pathogens. Among bacterial pathogens, *Salmonella abortus equi* [6] has long been recognized as the primary cause of equine abortion in China. Although viral pathogens such as *EHV-1* and *EVA* are also important causes of equine abortion, this study focuses on bacterial pathogens, primarily for the following reasons: First, bacterial abortion has been on the rise in Northwest China in recent years, and its prevention and control are closely related to antibiotic use. Second, there is a lack of systematic epidemiological data on bacterial pathogens (especially *S. abortus equi*, *Coxiella burnetii*, and *Chlamydia* spp.) in local horse populations. Third, the treatment of bacterial abortion often involves antibiotics, but drug resistance is becoming increasingly prominent, necessitating medication guidance based on genotype data.

*Chlamydia* spp. (particularly *C*. *abortus* and *C*. *psittaci*) is an obligate intracellular bacterium that can cause chronic infections and recurrent abortions [7]. Its detection rate in global equine abortion cases has been rising in recent years, as shown by reports such as R Akter [8] detection of *C*. *psittaci* in two aborted equine fetuses in Victoria, Akter, R [9] report on *C*. *psittaci* infection in pregnant mares and foals in Australia, and R Madison Ricard [10] detection of *C*. *abortus* in aborted equine fetuses in Western Canada.

*C. burnetii*, the causative agent of Q fever, is primarily transmitted via aerosols or tick vectors and is an important zoonotic pathogen. There is growing serological evidence of its association with abortion in equines, as demonstrated by studies such as Seo MG’s [11] report of *C. burnetii* infection in Korean horses through tick vectors, and Jaferi M’s [12] serological survey of *C. burnetii* in horses from eastern Iran. Additionally, *Brucella*, although not a primary host in horses, poses a significant zoonotic risk due to its high prevalence in ruminants and the increasing number of infection reports in equines in countries like Pakistan [13] and Nigeria [14]. Given the strong zoonotic potential of *Brucella* spp. [15,16], its potential threat in equine populations should not be overlooked.

Despite years of efforts to control *S. abortus equi* [17,18]-related equine abortion, the problem in northwest China has not been fundamentally resolved. This suggests that the pathogen spectrum for equine abortion may be more complex than traditionally understood. There could be other, less-recognized pathogens (such as *Chlamydia* spp. and *C. burnetii*) acting as primary or synergistic pathogenic factors, or the epidemiology of the pathogens may have shifted. However, there are significant gaps in current domestic research:

First, most studies remain at the level of clinical symptom descriptions and sporadic case reports, lacking large-scale, systematic epidemiological surveys covering major endemic areas and targeting multiple important bacterial pathogens. As a result, our understanding of the true distribution, prevalence intensity, and regional variations in these pathogens is limited.

Second, in farming practices, antibiotics are widely used for prevention and treatment [19]. However, due to varying management levels and the lack of proper medication guidance, antibiotic misuse and overuse are common. This inevitably generates selective pressure, leading to the development and spread of antibiotic resistance. Particularly concerning is the near-absence of research on antibiotic resistance monitoring in equine abortion-related pathogens, especially molecular-level screening for resistance genes, which has not been effectively carried out. This results in clinical treatment relying largely on experience rather than scientific evidence, leading to uncertain efficacy and exacerbating the resistance crisis.

Therefore, to effectively address the increasingly prominent issue of equine abortion in northwest China, a comprehensive study is urgently needed to fill the aforementioned knowledge gaps. This study aims to conduct a thorough cross-sectional survey of four key bacterial pathogens of equine abortion in the region—*Chlamydia* spp., *C. burnetii*, *Brucella* spp., and *S. abortus equi*. We will precisely assess their serological and molecular prevalence, analyze the associated risks related to geographical distribution, host age, gender, and species, and investigate mixed infection patterns in depth. More importantly, this study innovatively includes extensive screening for antibiotic resistance genes in positive samples, aiming to reveal, for the first time, the resistance gene profiles of these pathogens in equine populations. The study will construct a “pathogen-host-resistance” three-dimensional epidemiological map.

Ultimately, the findings of this study will provide crucial scientific evidence for developing evidence-based, targeted equine abortion prevention strategies and guiding rational clinical drug use. It will also have far-reaching significance for ensuring the sustainable development of China’s equine industry and safeguarding public health, particularly in relation to Q fever and brucellosis.

## 2. Materials and Methods

### 2.1. Study Area

**Figure 1:**
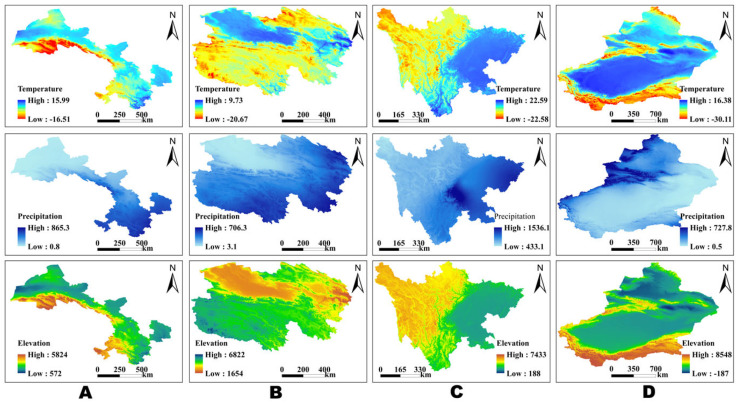
Geographical conditions of the sampling area.

This study selected the provinces in northwest China that have experienced large-scale equine abortions in recent years. These provinces include Gansu, Qinghai, Sichuan, and the Xinjiang Uygur Autonomous Region. Located primarily in northern China, these provinces account for over 50% of the total equine breeding volume in the country. The study area spans from 73°32′ to 108°46′ east longitude and from 26°03′ to 49°33′ north latitude, covering an area of approximately 3.329 million square kilometers. The annual average temperature in these regions ranges from −2 °C to 18 °C, with annual precipitation varying from 30 mm to 1000 mm. Additionally, the area encompasses a variety of terrains, including plateaus, plains, basins, deserts, and mountains (Figure 1).

Spatial patterns of mean annual temperature (top row, °C), mean annual precipitation (middle row, mm), and elevation (bottom row, m) across the four study provinces. 

Figure 1A: Gansu of mean annual temperature (top row, °C), mean annual precipitation (middle row, mm), and elevation (bottom row, m).

Figure 1B: Qinghai of mean annual temperature (top row, °C), mean annual precipitation (middle row, mm), and elevation (bottom row, m).

Figure 1C: Sichuan of mean annual temperature (top row, °C), mean annual precipitation (middle row, mm), and elevation (bottom row, m).

Figure 1D: Xinjiang of mean annual temperature (top row, °C), mean annual precipitation (middle row, mm), and elevation (bottom row, m).

Color scales are unified within each variable, with cooler-to-warmer colors indicating lower-to-higher values; north arrows and scale bars are shown for reference to facilitate cross-province comparisons.

### 2.2. Sample Size

The determination of the sample size was based on the calculation formula proposed by Zhang, H et al. (2023) [20], assuming a prevalence rate of 50%, a confidence level of 95%, and for detecting infection, with an accuracy setting of 5%. The formula used is as follows:

$${MOX}_{max}=z\sqrt{\frac{p(1-p)}{n}}$$

**Figure 2:**
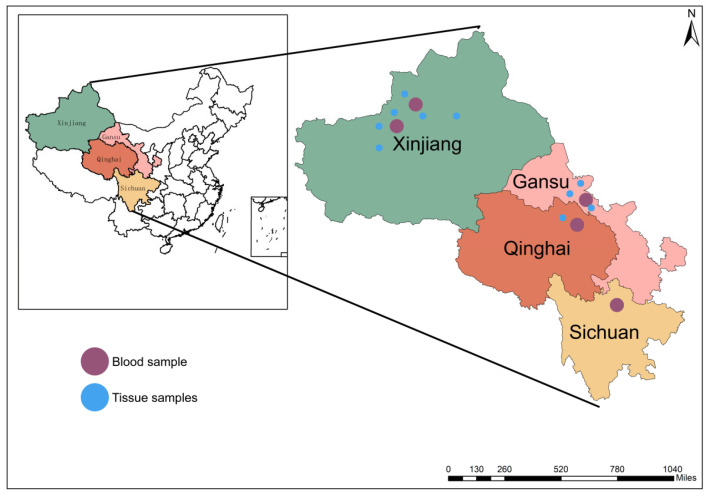
Sampling site map.

Here, n represents the required sample size, z is the z-value corresponding to the desired confidence level (for 95%, it can be expressed as 1.96), and p is the prevalence rate. Ultimately, we determined that at least 385 animals are needed to meet the maximum error of approximately ±5%. In order to enhance the accuracy of the test, a total of 800 blood samples were collected. However, due to various losses during transportation and other factors, we ultimately selected 508 blood samples and 24 samples from aborted fetuses. The maximum error can be reduced to approximately ±4.35% (Figure 2).

Study area and sampling locations. The inset map shows China and the four study provinces (Xinjiang, Gansu, Qinghai, Sichuan). The enlarged panels display the specific sites within each province: purple circles indicate blood-sample locations, and blue circles indicate tissue-sample locations. A north arrow and a scale bar (miles) are included; administrative boundaries are for reference only.

### 2.3. Herd Selection and Sample Collection

From October 2024 to August 2025, we conducted a cross-sectional survey in Gansu, Qinghai, Sichuan, and the Xinjiang Uygur Autonomous Region, with a minimum sample size of ≥100 equines per province. Farms were eligible if they met all of the following criteria: (1) Registered, large-scale equine operations; (2) The number of livestock being raised exceeds 500; (3) Veterinary services provided by official or on-site veterinarians; (4) Willingness to participate in the study; (5) The presence of an animal-welfare protection system.

### 2.4. Sampling

After obtaining consent from the farms, we followed animal protection and welfare regulations. Blood samples were collected from the selected horses via jugular venipuncture using blood collection tubes. Two tubes of blood were collected from each horse: one with an EDTA tube and the other with a plain collection tube. The collected blood was immediately sent to the laboratory. For the plain collection tube, after the blood coagulated at room temperature, serum was separated and stored at −20 °C for future use. The blood in the EDTA tubes was used for DNA extraction, and the extracted DNA was stored at −20 °C for later use.

For abortion tissue samples, we had previously reached an agreement with the farms to notify us immediately in the event of any abortion. After transferring the abortion tissue to a sterile environment, we used sterilized surgical knives and forceps to take tissue samples, primarily collecting the heart, liver, spleen, kidneys, amniotic fluid, and epidermal tissue of the aborted fetuses. The regular blood collection tubes (catalog number 2450003) and EDTA blood collection tubes (catalog number 2450111) used in this study were both sourced from Shandong HongYu Medical Technology Group Co., Ltd., Weihai, China.

Through a statistically standardized questionnaire, we finalized the use of 470 mare serum samples and 38 stallion serum samples. These samples correspond to 46 donkey serum samples, 462 equine serum samples, 63 serum samples from horses under 5 years old, 293 serum samples from horses aged 5 to 10 years, and 152 serum samples from horses over 10 years old (Detailed information can be found in Table A1—Sample Classification Information).

### 2.5. Serological Diagnosis

For serological testing, all samples were analyzed using commercially available ELISA kits. *Chlamydia* spp. and *C. burnetii* were tested using ELISA kits from Shanghai Keaibo Biotech Co., Ltd., Shanghai, China, with catalog numbers CB10603-Sp and CB10090-Gt, respectively. *Brucella* spp. was tested using a commercial ELISA kit from Shijiazhuang Shengbo Biotech Co., Ltd., Shijiazhuang, China, with catalog number BRU-I2P. *S. abortus equi* was tested using a commercial cELISA kit from Guosheng Biotech Co., Ltd., Harbin, China, with catalog number NWMLC142C. All serological tests were conducted strictly according to the instructions provided with the commercial kits, and the OD450 values were measured using a microplate reader at the end of the process. The ELISA kit used in this study showed sensitivities and specificities of 92% and 95% for *Chlamydia* spp., 89% and 94% for *C. burnetii*, 90% and 96% for *Brucella* spp. and 90% and 96% for *S. abortus equi*, respectively.

### 2.6. DNA Extraction and Real-Time PCR Detection

We used the DNA extraction kit for blood/cells/tissues genomic DNA, produced by Tian Gen (China) Company, Beijing, China, catalog number DP304-03. Following the manufacturer’s instructions, we extracted DNA from blood and abortion tissue samples.

Subsequently, primers and probes targeting the *Chlamydia* spp. gene, the IS1111a gene of *C. burnetii*, the bcsp31 gene of *Brucella* spp., and the FljB gene of *S. abortus equi* were designed. All primers were synthesized by GenScript, Sydney, Australia, and the enzyme reagents were provided by Vazyme Biotech Co., Ltd., Nanjing, China (The primers and procedures used in real-time PCR can be found in Table A2: “Real-time PCR partial target gene sequence” and Table A3: “Real-time PCR reaction procedure”.) The Real-time PCR method had both sensitivity and specificity exceeding 99%.

### 2.7. The Methods for Detecting Antibiotic Resistance Genes

To investigate the presence of antibiotic resistance genes, we used the Antibiotic Resistance Detection Panel (RUO) from A2K Scientific to detect resistance genes in Real-Time PCR positive samples. This kit includes 29 resistance genes, corresponding to multiple classes of antibiotics. (Antibiotic resistance genes correspond to antibiotics as shown in Table A4).

### 2.8. Ethics

All the animal experiments in this study have been approved by the Animal Ethics Committee of Lanzhou Veterinary Research Institute of Chinese Academy of Agricultural Sciences (Approval Number: LVRIAEC-2020-058) (Approval Date: May 2020). All the animal experiments during the study (including blood collection from the animals) were conducted in accordance with the relevant regulations and guidelines on animal ethics in China. The collection of blood samples was carried out after obtaining the informed consent of the owners.

## 3. Results

### 3.1. The Detection Results of Chlamydia spp.

A total of 15 farms in northwest China participated in this study, including 12 horse farms (80%) and 3 donkey farms (20%), with a total of 508 blood samples from animals collected.

Through ELISA testing of 508 serum samples, the overall positivity rate for *Chlamydia* spp. infection antibodies was 18.90%. The antibody positivity rate was highest in Sichuan (29.17%), followed by Xinjiang (21.79%), Qinghai (12.93%) and Gansu (7.64%). Donkeys had a significantly higher antibody positivity rate of 28.26%, compared to horses, which had a rate of 17.97%. Among different age groups, animals ≤ 5 years had an antibody positivity rate of 20.63%, animals aged 5.1–10 years had 17.75%, and animals > 10 years had 20.39%. The antibody positivity rate was similar between females (18.94%) and males (18.42%) (Table 1).

The overall positivity rate for *Chlamydia* spp. by Real-Time PCR was 14.17%. Xinjiang had the highest positivity rate at 22.00%, followed by Qinghai at 12.93%, Sichuan at 8.33%, and Gansu at the lowest rate of 6.25%. The positivity rate in donkeys was 21.73%, higher than in horses, which had a rate of 13.41%. Among different age groups, animals ≤ 5 years had a positivity rate of 12.69%, animals aged 5.1–10 years had 13.31%, and animals > 10 years had 16.67%. The positivity rates for females (14.25%) and males (13.16%) were similar (Table 2).

The positivity rate for Real-Time PCR was lower than that of the ELISA test. Donkeys had higher positivity rates in both ELISA (28.26%) and PCR (21.73%) compared to horses (ELISA, 17.97%, PCR, 13.41%). There were significant differences in antibody positivity rates across provinces (*p* < 0.01), with ELISA showing the highest positivity rate in Sichuan (29.17%) and PCR showing the highest in Xinjiang (22.00%). This suggests that *Chlamydia* spp. infection is more likely to occur in Xinjiang and Sichuan, with donkeys showing higher susceptibility than horses.

### 3.2. The Detection Results of C. burnetii

Through ELISA testing of 508 serum samples, the overall antibody positivity rate for *C. burnetii* infection was 28.94%. Sichuan had the highest antibody positivity rate at 35.42%, followed by Xinjiang at 34.50%, Qinghai at 25.86%, and Gansu at the lowest rate of 21.53%. Donkeys had a higher antibody positivity rate of 32.61%, compared to horses, which had a rate of 28.57%. Animals aged 5.1–10 years had a significantly higher antibody positivity rate of 34.47%, compared to animals ≤ 5 years (26.98%) and >10 years (19.08%) (*p* < 0.01). The antibody positivity rate in females was 28.94%, and in males, it was 28.95% (Table 3).

The overall positivity rate for *C. burnetii* by Real-Time PCR was 15.35%. Xinjiang had the highest positivity rate at 18.00%, followed by Gansu at 13.19%, Sichuan at 16.67%, and Qinghai at the lowest rate of 12.93%. Donkeys had a positivity rate of 13.04%, while horses had a slightly higher rate of 15.58%. Animals ≤ 5 years had a significantly higher positivity rate of 26.98%, compared to animals aged 5.1–10 years (15.70%) and >10 years (9.87%). The positivity rate in females (8.09%) was lower than in males (21.05%) (Table 4).

The positivity rate for Real-Time PCR was lower than that for ELISA testing. Both Real-Time PCR and ELISA testing indicated that horses ≤ 10 years old were most susceptible to *C. burnetii*. This suggests that *C. burnetii* infections are more likely to occur in Xinjiang and Sichuan, with younger and adult horses being more susceptible.

### 3.3. The Detection Results of Brucella spp.

The ELISA detection results for *Brucella* spp. showed an overall antibody positivity rate of 0.78%. Positive serum samples were only detected in Gansu Province, with 4 positive cases, and no positive cases were found in other provinces (Table 5). However, subsequent PCR testing of these samples did not yield any positive results. This suggests that the horses have either been previously infected or developed antibodies through contact with other animals or humans infected with *Brucella* spp.

### 3.4. The Detection Results of S. abortus equi

Through ELISA testing of 508 serum samples, the overall antibody positivity rate for *S. abortus equi* infection was 35.03%. Xinjiang had the highest antibody positivity rate at 44.50%, followed by Qinghai at 31.90%, Gansu at 28.47%, and Sichuan at the lowest rate of 22.92%. Donkeys had a significantly higher antibody positivity rate of 89.13%, compared to horses, which had a rate of 29.65%. Animals aged 5.1–10 years had an antibody positivity rate of 34.81%, animals ≤ 5 years had 22.22%, and animals > 10 years had 40.79%. The antibody positivity rate in females (36.60%) was higher than in males (15.79%) (Table 6).

The overall positivity rate for *S. abortus equi* by Real-Time PCR was 22.03%. Xinjiang had the highest positivity rate at 25.50%, followed by Gansu at 22.92%, Qinghai at 21.55%, and Sichuan at the lowest rate of 16.67%. Donkeys had a significantly higher positivity rate of 76.09%, compared to horses, which had a rate of 17.75%. Animals ≤ 5 years had a positivity rate of 9.52%, animals aged 5.1–10 years had 24.91%, and animals > 10 years had 25.00%. The positivity rate in females (22.55%) was lower than in males (28.95%) (Table 7).

The positivity rate for Real-Time PCR was lower than that for ELISA testing. Both Real-Time PCR and ELISA tests indicated that Xinjiang is more prone to *S. abortus equi* outbreaks, with donkeys being more susceptible to the infection than horses.

### 3.5. Detection Results of Abortion Tissue Samples and Mixed Infections

**Figure 3:**
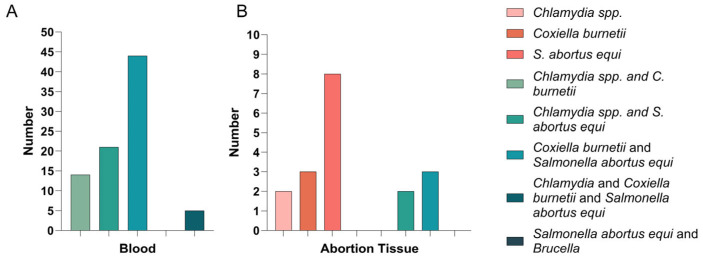
Pathogen Co-infection Patterns. (**A**): The number of mixed infections in PCR-positive blood samples. The bars represent different pathogens or their combinations, with the vertical axis indicating the number of positive individuals. (**B**): The number of single/mixed infections in PCR-positive abortion tissue samples. Pathogens involved include *Chlamydia* spp., *C. burnetii*, *S. abortus equi*, and *Brucella* spp. Composite labels indicate co-detection of multiple pathogens.

In the tissue samples, the highest detection rate was found for *S. abortus equi* (8), followed by *C. burnetiid* (3), and *Chlamydia* spp. (2), while *Brucella* spp. was not detected in the tissue samples (Figure 3B). In the blood detection corresponding to the mares from which abortion tissues were collected, we found that Real-Time PCR detected *S. abortus equi* infection in 8 samples, *C. burnetii* infection in 3 samples, and *Chlamydia* spp. infection in 3 samples. ELISA testing revealed that 11 samples were infected with *S. abortus equi*, 6 samples with *C. burnetii*, and 3 samples with *Chlamydia* spp.

We also conducted a statistical analysis of mixed infections. In both Real-Time PCR and ELISA tests, 44 samples were co-infected with *C. burnetii* and *S. abortus equi*, 21 samples were co-infected with *Chlamydia* spp. and *S. abortus equi*, 14 samples were co-infected with *Chlamydia* spp. and *C. burnetiid* (Figure 3). In tissue samples, 3 samples were co-infected with *C. burnetiid* and *S. abortus equi*, 2 samples were co-infected with *Chlamydia* spp. and *C. burnetiid* (Figure 3B).

### 3.6. Detection of Antibiotic Resistance Genes

**Figure 4:**
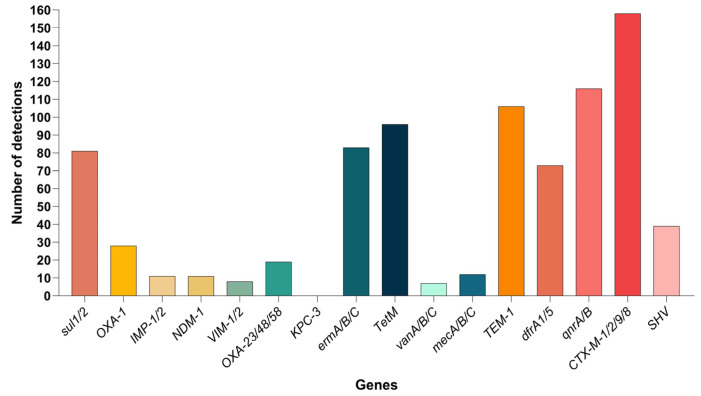
Antibiotic resistance gene detection. The count of antibiotic resistance genes detected in PCR-positive DNA samples. The vertical axis represents the count of detected resistance genes.

After excluding mixed infections, we performed Real-Time PCR detection of antibiotic resistance genes in a total of 196 positive DNA samples (including both tissue and blood samples). The most commonly detected resistance gene was *CTX-M-1/2/9/8*, with 158 detections, followed by the *TEM-1* gene with 106 detections, *TetM* with 96 detections, *ermA/B/C* with 83 detections, *qnrA/B* with 116 detections, *sul1/2* with 81 detections, *dfrA1/5* with 73 detections, *SHV* with 39 detections, *OXA-1* with 28 detections, and *OXA-23/48/58* with 19 detections. The *mecA/B/C* genes were detected in 12 samples, *IMP-1/2* in 11, *NDM-1* in 11, *VIM-1/2* in 8, *vanA/B/C* in 7, and *KPC-3* was not detected. Multiple samples often showed the presence of several resistance genes simultaneously (Figure 4). In the blood detection corresponding to the mares from which abortion tissues were collected, we mainly detected the antibiotic resistance genes *CTX-M* (n = 18), *qnrA/B* (n = 17), *sul1/2* (n = 11), and *erm* (n = 5). Moreover, *CTX-M* and *qnrA/B* were always detected together, and no other resistance genes were detected.

From a statistical perspective, the following resistance genes were detected: 197 instances of ESBL (Extended Spectrum Beta-Lactamase) resistance genes, 134 instances of Penicillin resistance genes, 116 instances of Fluoroquinolone resistance genes, 96 instances of Tetracycline resistance genes, 83 instances of Macrolide/Lincosamide/Streptogramin resistance genes, 81 instances of Sulfonamide resistance genes, 73 instances of Trimethoprim resistance genes, 49 instances of Carbapenem resistance genes, 12 instances of Methicillin/Penicillin resistance genes, 7 instances of Vancomycin resistance genes.

Among the 196 PCR-positive samples, the most common gene combination was *CTX-M + TEM-1* (n = 89), followed by *CTX-M + qnrA/B* (n = 67), suggesting that *ESBL* and *PMQR* genes often coexist in the same strain or sample. Combined with the PCR-positive results for the pathogens, it can be inferred that the ESBL gene mainly originated from *S. abortus equi* and *C. burnetii*-positive samples, while the erm gene may be related to *Chlamydia* spp.

This data indicates that resistance to ESBL, Penicillin, Fluoroquinolones, Tetracyclines, Macrolide/Lincosamide/Streptogramin, Sulfonamides, Trimethoprim, and Carbapenems is a significant issue, with widespread resistance detected across multiple antibiotic classes.

## 4. Discussion

This study primarily reports the prevalence of four bacterial pathogens that can cause equine abortion in the provinces of northwest China, where large-scale equine abortion outbreaks have occurred in recent years. It also investigates the presence of antibiotic resistance genes in the positive DNA samples to assess their resistance profiles. The aim is to provide scientifically based recommendations for the treatment of equine abortion.

**Figure 5:**
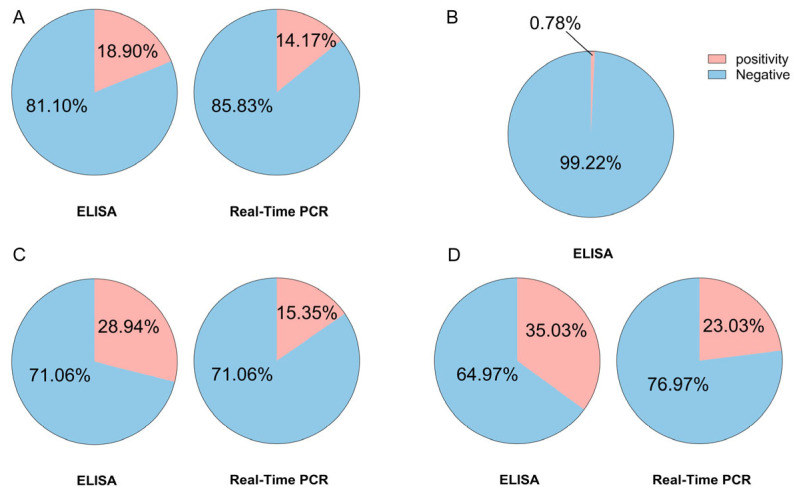
Serological and molecular prevalence of four bacterial pathogens associated with equine abortion in northwest China. (**A**) *Chlamydia* spp., (**B**) *Brucella* spp., (**C**) *Coxiella burnetii*, and (**D**) *S. abortus equi.* For each pathogen, the left pie chart shows ELISA results and the right pie chart shows real-time PCR results (no PCR-positive samples were detected for Brucella spp., so only ELISA results are presented). Pink sectors indicate positive samples and blue sectors indicate negative samples, with the corresponding percentages labeled inside each pie (n = 508).

Through this cross-sectional study, both ELISA and Real-Time PCR consistently pointed to *S. abortus equi* as the most frequently detected pathogen (Figure 5), with the highest positivity rate observed in Xinjiang (serological positivity rate of 44.50%, Real-Time PCR positivity rate of 25.50%) (Table 6 and Table 7). *C. burnetii* and *Chlamydia* spp. showed moderate prevalence in multiple provinces, suggesting that these pathogens may persist in the host for extended periods. Even without obvious symptoms, they may trigger new infections when the host is re-exposed. Although the overall positivity for *Brucella* spp. was low, seropositive animals were identified in our testing, and we will continue longitudinal follow-up surveillance.

Both serological and tissue samples consistently indicated that mixed infections (i.e., *S. abortus equi + C. burnetii*) are not uncommon (Figure 3), with this combination also detected in fetal tissues. This aligns with the observation that in high-risk abortion cases, the primary pathogenic bacteria, along with other potentially problematic bacteria, act together, leading to placental inflammation and increasing the risk of abortion. Multiple studies have shown that co-infections may enhance pathogenicity through synergistic mechanisms. A similar mechanism may be involved in equine abortion, which requires further experimental validation [21,22].

Compared to other recent studies [23], the positivity rate for *S. abortus equi* in Xinjiang (serological positivity rate of 44.50%, Real-Time PCR positivity rate of 25.50%) (Table 6 and Table 7) was slightly elevated, but the overall level remained similar. While the antibody positivity rate for *C. burnetii* was relatively high in Xinjiang (Table 3), it did not reach the 57% positivity rate reported by Jixu Li [24], which may be due to differences in our sampling locations. Moreover, there is currently no clear epidemiological survey report for *Chlamydia* spp. and *Brucella* spp. in China.

In this study, we found that the serological positivity rate (18.90%, 28.94, 0.87%, 35.03%) (Table 1, Table 3, Table 5 and Table 6) was generally higher than the Real-Time PCR positivity rate (14.17%, 15.35%, 0, 23.03%) (Table 2, Table 4 and Table 7), reflecting the differences between immunological and molecular biological testing. A higher serological positivity rate typically indicates that the animal may have experienced a past infection that has already been resolved, and this infection may not have triggered obvious clinical symptoms, resulting in the formation of antibody-mediated immune memory. As described in articles by Muroni G [25] and Ana L García-Pérez [26], the lower positivity rate observed by Real-Time PCR may be related to intermittent or low-level pathogen shedding. Given that the amount and duration of pathogen shedding in the host can fluctuate, especially in cases of chronic or subclinical infections, Real-Time PCR’s sensitivity may be insufficient to detect low concentrations of bacterial DNA.

Therefore, serological testing can reflect past infection in the equine population, while Real-Time PCR provides more direct evidence of current infection. However, the two methods have different sensitivities and limitations in reflecting the disease status. The ELISA positivity rate is generally higher than that of PCR, possibly due to the following factors: First, ELISA detects antibodies, reflecting past or persistent infection; second, PCR detects pathogen DNA, which is affected by the bacterial load in the sample and the timing of sampling; third, cross-reactivity between ELISA and other pathogens cannot be ruled out, especially in samples that are serologically positive for *Brucella* spp. but PCR negative. This difference suggests that combining serological and Real-Time PCR testing in epidemiological monitoring may be more comprehensive and effective, particularly in contexts where low-level shedding or chronic infection cannot be completely ruled out.

The study found that *C. burnetii* and *S. abortus equi* exhibited significantly higher Real-Time PCR or serological positivity rates in equine aged 5 to 10 years (serological positivity rate of 34.47%, 34.81%, Real-Time PCR positivity rate of 15.70%, 24.91%) (Table 3, Table 4, Table 6 and Table 7). This phenomenon may be related to the reproductive activities of equine in this age group, particularly the frequent mating, pregnancy, and stress responses during parturition and transport, which are closely associated with sexual maturity. The immune system of these equine may generate different immune responses when exposed to continuous pathogen infection. This variation in immune responses, combined with increased opportunities for infection, may contribute to their susceptibility. Additionally, age may influence immune system responsiveness, especially regarding immune tolerance and recovery, which could make equine in this age group more prone to persistent or chronic infections when exposed to pathogens. This situation is also supported by studies from James Mutiiria Kithuka [27] and Zhanhai Mai [23].

Furthermore, the study revealed that donkeys had a significantly higher positivity rate than horses (Table 1, Table 2 and Table 3, 6 and 7), reflecting species-specific differences in disease susceptibility, immune responses, and pathogen transmission routes. As highlighted in Manling Zhu’s [28] article, the herd of donkeys shows latent infection. Therefore, mixed-species farming of donkeys and horses may act as an amplifier for disease transmission, especially under conditions of high-density farming and cross-species transmission. This suggests that species susceptibility and behavioral ecological differences under mixed-species farming conditions may enhance pathogen transmission between subgroups. Additionally, farming management practices (such as pen density, bedding, and manure disposal) and transport stress may act as key triggers.

In the analysis of 196 PCR-positive DNA samples, excluding mixed infections, the detected antibiotic resistance genes revealed a pattern centered on ESBL (Extended Spectrum Beta-Lactamase), with coexisting resistance to multiple antibiotic classes: ESBL and broad-spectrum cephalosporin-related genes were the most commonly detected (197 instances), followed by penicillin resistance (134), fluoroquinolone resistance (116), tetracycline resistance (96), MLS resistance (83), sulfonamide resistance (81), trimethoprim resistance (73), and carbapenem resistance (49). These results indicate a serious resistance situation for ESBL, penicillin, fluoroquinolones, tetracyclines, MLS, sulfonamides, and trimethoprim.

This characteristic of drug resistance is in clear agreement with the practice in clinical settings where third-generation cephalosporins or fluoroquinolones are selected based on experience. The widespread detection of CTX-M, TEM, SHV, and qnr suggests that continuing to rely on these two antibiotic classes as empirical first-line treatments will likely lead to higher treatment failure rates and increased selective pressure. The high frequency of tetM and erm gene detection suggests that traditional treatment regimens relying on tetracyclines or macrolides for pathogens like *Chlamydia* spp. and *C. burnetii* may become less effective.

From a genetic perspective, ESBL genes and PMQR (Plasmid-mediated quinolone resistance) [29], along with sul/dfr genes, are often co-located on plasmids/integrons, facilitating horizontal gene transfer between Enterobacteriaceae and Salmonella, thereby maintaining and spreading the “multi-locus resistance” ecosystem. Although carbapenemase and mec genes were less common, their presence serves as an important warning: once these key human-associated resistance genes enter the farm-environment-human cycle, they significantly increase the risk of cross-species transmission and clinical rebound. The relatively low detection of glycopeptide resistance genes confirms the effectiveness of restrictive drug policies.

Based on this evidence, the study suggests an evidence-based “Sample-Test-Treat” approach and tiered management strategy: Diagnosis first: Sample collection should precede treatment during abortion or reproductive tract events, with both culture + phenotypic susceptibility testing and genotypic resistance profiling performed simultaneously. Third-generation cephalosporins and fluoroquinolones should be downshifted from empirical first-line to second-line/rescue treatments based on evidence. De-escalation and Narrow-spectrum Use: After test results return, narrow the coverage and limit treatment duration to avoid mass, prophylactic, or prolonged use of tetracyclines/macrolides. Pathogen-specific management: For high-risk *C. burnetii* farms, focus on management of parturition/abortion sites, environmental sanitation, and ventilation, with vaccines and targeted treatments when necessary. For suspected *Chlamydia* spp.-related reproductive failure, emphasize isolation, quarantine, and individualized treatment to avoid “mass treatment.” Establish local antibiograms and annual AMR reports: Use monitoring data to dynamically update treatment guidelines and standard operating procedures (SOPs). One Health integration: Share monitoring information on Q fever, Salmonella, ESBL, PMQR, and other relevant pathogens with cattle and sheep farms and local disease control departments to block the flow of resistance genes across farms, hospitals, and communities.

Overall, the combination of “high ESBL and PMQR burden + multi-drug co-resistance + sporadic detection of key human-associated resistance genes” revealed in this study highlights the structural mismatch between current clinical empirical practices and the actual antibiotic resistance ecosystem. A comprehensive strategy, centered on evidence-based treatment, restricted use of key drugs, non-pharmacological interventions, and cross-departmental coordination, offers a viable path to reducing abortion burdens and curbing the spread of resistance.

This study has several strengths. First, it covers multiple provinces with a large sample size, providing strong representativeness and reflecting the distribution of equine abortion pathogens and their resistance characteristics across different regions. Second, the study employed a combination of ELISA and Real-Time PCR for detecting pathogens in abortion tissue samples. By utilizing multiple detection techniques in parallel, the accuracy and reliability of the results were enhanced. Furthermore, we incorporated antibiotic resistance gene investigation into the epidemiological survey, establishing a “pathogen-host-resistance” three-dimensional framework. This multidimensional approach offers new insights into the pathogenesis and resistance profiles of equine abortion, facilitating a deeper understanding of the microbial characteristics and resistance mechanisms involved.

The main innovation of this study lies in its systematic epidemiological survey of *S. abortus equi* (serological positivity rate of 35.03%, Real-Time PCR positivity rate of 23.03%), *C. burnetiid* (serological positivity rate of 28.94%, Real-Time PCR positivity rate of 15.35%), *Chlamydia* spp. (serological positivity rate of 18.90%, Real-Time PCR positivity rate of 14.17%), and *Brucella* spp. (serological positivity rate of 0.78%, Real-Time PCR positivity rate of 0) (Table 1, Table 2, Table 3, Table 4, Table 5, Table 6 and Table 7) related to equine abortion in China. It also connects the AMR gene profiles with the mismatch in clinical drug use, establishing an integrated “pathogen-host-resistance” evaluation system. Additionally, it proposes farm SOPs based on risk stratification and One Health collaborative monitoring. The integrated evidence presented in this study provides a practical path for evidence-based drug use and region-specific control strategies.

However, there are some limitations to the study. First, as a cross-sectional design was employed, causal relationships cannot be definitively established, so further longitudinal studies are needed to clarify the causal link between abortion and pathogens. Second, the study did not include pathogen isolation and whole-genome sequencing (WGS), meaning that the resistance loci and transmission chains of the pathogens remain unclear, limiting the in-depth understanding of resistance mechanisms. Additionally, although abortion tissue samples were collected, the sample size was relatively small. These limitations provide guidance for future research directions. Future studies could include longitudinal research, such as pathogen isolation, whole-genome sequencing, and mechanism studies, to further improve the findings.

To effectively control the occurrence and spread of equine abortion, this study proposes a series of control and management measures. First, immediate isolation of mares during parturition and abortion should be implemented, and the collection and safe disposal of abortion tissues and secretions should be standardized to prevent the spread of pathogens in the environment. Additionally, it is recommended to strengthen personal protective measures for staff to reduce the risk of infection.

Furthermore, in terms of breeding management, it is advised to implement separate feeding for pregnant mares, replacement mares, and different species. The time spent in high-density mixed-group housing should be minimized to reduce opportunities for pathogen transmission.

In terms of diagnosis and monitoring, the study recommends incorporating *S. abortus equi*, *C. burnetii*, and *Chlamydia* spp. into the reproductive disorder screening system (including ELISA and Real-Time PCR tests). It also suggests conducting quarterly rolling monitoring of at-risk groups with recurrent abortions to ensure the timely detection and management of potential infection risks.

For medication management, the appropriate treatment drugs should be selected based on pathogen isolation combined with susceptibility testing or resistance gene profiling. This will help avoid the indiscriminate use of cephalosporins and fluoroquinolones. The study advocates for a treatment strategy focused on supportive therapy and biosecurity measures, aimed at reducing drug misuse and addressing resistance issues. Based on the high-frequency ESBL genes (CTX-M, TEM) and PMQR genes (qnrA/B) detected in this study, it is recommended to avoid using third-generation cephalosporins and fluoroquinolones as empirical first-line choices before obtaining the results of drug susceptibility tests. With the support of antimicrobial resistance gene detection, amoxicillin-clavulanic acid, tigecycline, or polymyxin can be considered as alternative options, and the duration of treatment should be strictly limited.

Finally, from a One Health perspective, it is recommended to strengthen collaboration between equine farms, livestock farms, and local disease control departments, sharing monitoring information on abortion and diseases such as Q fever and brucellosis. This will help reduce the risks of cross-species transmission and occupational exposure. This comprehensive control strategy not only helps reduce the occurrence of equine abortion but also effectively controls the spread of pathogens, ultimately improving overall public health levels.

## 5. Conclusions

This study conducted an epidemiological survey and antibiotic resistance gene analysis of *S. abortus equi*, *C. burnetii*, *Chlamydia* spp., and *Brucella spp*. in equine populations across multiple provinces in China, revealing the heterogeneous distribution of these pathogens by region, species, and age. The results show that *S. abortus equi* is the primary pathogen, with *C. burnetii* and *Chlamydia* spp. being co-endemic, while no active *Brucella* spp. infections were detected, although caution is still advised. Regarding antibiotic resistance, higher detection rates were observed for cephalosporins, fluoroquinolones, penicillin, sulfonamides, and tetracyclines, reflecting a mismatch between empirical drug use and the resistance ecology. It is recommended that clinical treatment be guided by pathogen culture, susceptibility testing, and genotype resistance profiles, avoiding the indiscriminate use of cephalosporins and fluoroquinolones. The study also showed higher positivity rates in equines aged 5 to 10 years and in donkeys, indicating that age and species-based segregation, isolation, and breeding unit management should be implemented. From a “One Health” perspective, strengthening joint monitoring with cattle and sheep herds and implementing cross-species control measures is crucial. Standardizing the handling of parturition/abortion sites and improving personal protection are key strategies, with precise monitoring and rational drug use at the core, to reduce the burden of abortion and the risk of antibiotic resistance. This study provides crucial evidence and pathways for managing equine abortion pathogens and antibiotic resistance in China, laying the foundation for future research and practice. Future research will focus on pathogen isolation, whole-genome sequencing, and the mechanisms of horizontal gene transfer of resistance.

## Figures and Tables

**Table 1:** ELISA results of *Chlamydia* spp. and related analysis.

Variable	Category	Pos./Tested	Prev.%	95%Cl	*p*-Value
Location	GanSu	11/144	7.64	(3.65, 11.63)	X2 = 28.719 *p* < 0.01
	QingHai	15/116	12.93	(7.01, 18.85)	
	XinJiang	56/200	21.79	(21.79, 34.21)	
	SiChuan	14/48	29.17	(16.38, 41.96)	
Species	Donkey	13/46	28.26	(15.35, 41.17)	X2 = 2.893 *p* = 0.089
	Horse	83/462	17.97	(14.50, 21.44)	
Age	≤5	13/63	20.63	(10.75, 30.51)	X2 = 0.599 *p* = 0.741
	5.1–10 years	52/293	17.75	(13.38, 22.12)	
	>10 years	31/152	20.39	(14.03, 26.75)	
Sex	Female	89/470	18.94	(15.37, 22.51)	X2 = 0.006 *p* = 0.938
	Male	7/38	18.42	(6.30, 30.54)	
Total		96/508	18.90	(15.48, 22.67)	

**Table 2:** Real-Time PCR results of *Chlamydia* spp. and related analysis.

Variable	Category	Pos./Tested	Prev.%	95%Cl	*p*-Value
Location	GanSu	9/144	6.25	(0.03, 0.12)	X2 = 18.99 *p* < 0.01
	QingHai	15/116	12.93	(0.07, 0.20)	
	XinJiang	44/200	22.00	(0.16, 0.28)	
	SiChuan	4/48	8.33	(0.02, 0.17)	
Species	Donkey	10/46	21.73	(0.11, 0.36)	X2 = 1.75 *p* = 0.186
	Horse	62/462	13.41	(0.10, 0.17)	
Age	≤5	8/63	12.69	(0.06, 0.23)	X2 = 0.94 *p* = 0.626
	5.1–10 years	39/293	13.31	(0.10, 0.18)	
	>10 years	25/152	16.67	(0.11, 0.23)	
Sex	Female	67/470	14.25	(0.11, 0.18)	X2 = 0.0264 *p* = 0.871
	Male	5/38	13.16	(0.04, 0.24)	
Total		72/508	14.17	(0.11, 0.18)	

**Table 3:** ELISA results of *C. burnetii* and related analysis.

Variable	Category	Pos./Tested	Prev.%	95%Cl	*p*-Value
Location	GanSu	31/144	21.53	(15.29, 28.94)	X2 = 8.368 *p* = 0.039
	QingHai	30/116	25.86	(18.36, 34.56)	
	XinJiang	69/200	34.50	(27.90, 41.57)	
	SiChuan	17/48	35.42	(22.38, 50.20)	
Species	Donkey	15/46	32.61	(19.83, 47.54)	X2 = 0.332 *p* = 0.565
	Horse	132/462	28.57	(24.54, 32.87)	
Age	≤5	17/63	26.98	(16.80, 39.35)	X2 = 11.664 *p* < 0.01
	5.1–10 years	101/293	34.47	(29.12, 40.09)	
	>10 years	29/152	19.08	(13.19, 26.17)	
Sex	Female	136/470	28.94	(24.91, 33.19)	X2 = 0.000 *p* = 0.999
	Male	11/38	28.95	(15.77, 45.33)	
Total		147/508	28.94	(24.89, 33.19)	

**Table 4:** Real-Time PCR results of *C. burnetii* and related analysis.

Variable	Category	Pos./Tested	Prev.%	95%Cl	*p*-Value
Location	GanSu	19/144	13.19	(0.08, 0.19)	X2 = 2.18 *p* = 0.536
	QingHai	15/116	12.93	(0.07, 0.19)	
	XinJiang	36/200	18.00	(0.13, 0.24)	
	SiChuan	8/48	16.67	(0.06, 0.27)	
Species	Donkey	6/46	13.04	(0.43, 0.23)	X2 = 0.06 *p* = 0.809
	Horse	72/462	15.58	(0.12, 0.19)	
Age	≤5	17/63	26.98	(0.16, 0.38)	X2 = 10.10 *p* < 0.01
	5.1–10 years	46/293	15.70	(0.12, 0.20)	
	>10 years	15/152	9.87	(0.05, 0.15)	
Sex	Female	70/470	8.09	(0.06, 0.11)	X2 = 0.61 *p* = 0.436
	Male	8/38	21.05	(0.08, 0.34)	
Total		78/508	15.35	(0.12, 0.18)	

**Table 5:** ELISA results of *Brucella* spp. and related analysis.

Variable	Category	Pos./Tested	Prev.%	95%Cl	*p*-Value
Location	GanSu	4/144	2.08	(0.43, 6.00)	X2 = 10.19 *p* = 0.017
	QingHai	0/116	0.00	(0.00, 3.16)	
	XinJiang	0/200	0.00	(0.00, 7.40)	
	SiChuan	0/48	0.00	(0.00, 7.40)	
Species	donkey	0/46	0.00	(0.00, 7.70)	X2 = 0.401 *p* = 0.526
	horse	4/462	0.87	(0.24, 2.21)	
Age	≤5	1/63	1.59	(0.04, 8.51)	X2 = 1.932 *p* = 0.381
	5.1–10 years	3/293	1.02	(0.21, 2.97)	
	>10 years	0/152	0.00	(0.00, 2.41)	
Sex	Female	4/470	0.85	(0.23, 2.17)	X2 = 0.326 *p* = 0.568
	Male	0/38	0.00	(0.00, 9.26)	
Total		4/508	0.78	(0.21, 1.99)	

**Table 6:** ELISA results of *S. abortus equi* and related analysis.

Variable	Category	Pos./Tested	Prev.%	95%Cl	*p*-Value
Location	GanSu	41/144	28.47	(21.42, 36.35)	X2 = 14.195 *p* = 0.003
	QingHai	37/116	31.90	(23.69, 41.04)	
	XinJiang	89/200	44.50	(37.45, 51.72)	
	SiChuan	11/48	22.92	(12.26, 37.03)	
Species	Donkey	41/46	89.13	(76.43, 96.43)	X2 = 65.017 *p* < 0.01
	Horse	137/462	29.65	(25.56, 33.98)	
Age	≤5	14/63	22.22	(12.97, 34.21)	X2 = 6.761 *p* = 0.034
	5.1–10 years	102/293	34.81	(29.41, 40.52)	
	>10 years	62/152	40.79	(33.03, 48.92)	
Sex	Female	172/470	36.60	(32.18, 41.19)	X2 = 6.687 *p* = 0.010
	Male	6/38	15.79	(6.05, 31.34)	
Total		178/508	35.03	(30.83, 39.39)	

**Table 7:** Real-Time PCR results of *S. abortus equi* and related analysis.

Variable	Category	Pos./Tested	Prev.%	95%Cl	*p*-Value
Location	GanSu	33/144	22.92	(0.16, 0.30)	X2 = 1.93 *p* = 0.587
	QingHai	25/116	21.55	(0.15, 0.29)	
	XinJiang	51/200	25.5	(0.20, 0.32)	
	SiChuan	8/48	16.67	(0.06, 0.27)	
Species	Donkey	35/46	76.09	(0.63, 0.87)	X2 = 77.06 *p* < 0.001
	Horse	82/462	17.75	(0.14, 0.21)	
Age	≤5	6/63	9.52	(0.03, 0.17)	X2 = 7.40 *p* = 0.025
	5.1–10 years	73/293	24.91	(0.20, 0.30)	
	>10 years	38/152	25.00	(0.18, 0.32)	
Sex	Female	106/470	22.55	(0.19, 0.26)	X2 = 0.49 *p* = 0.484
	Male	11/38	28.95	(0.16, 0.45)	
Total		117/508	23.03	(0.19, 0.26)	

## Data Availability

For detailed data, please contact the author directly.

## Appendix A

**Table A1:** Sample classification information.

Location	Sex		Species		Age			Total	
	Female	Male	Donkey	Horse	≤5	5.1–10 Years	>10 Years	Blood	Aborted Tissue
GanSu	134	10	25	119	27	63	54	144	5
QingHai	108	8	6	110	11	66	39	116	1
XinJiang	180	20	10	190	22	138	40	200	18
SiChuan	48	0	5	43	3	26	19	48	0

**Table A2:** Real-time PCR partial target gene sequence.

	Primers	Sequence
*Chlamydia* spp.	Chla-F	GCAACTGACACTAAGTCGGCTACA
	Chla-R	ACAAGCATGTTCAATCGATAAGAGA
	Chla-P	FAM-TAAATACCAACGAATGGCAAGTTGGTTTAGCG-TAMRA
*C. burnetii*	ISlllla-F	TGGGCTGCGTGGTGATG
	ISlllla-R	TGACGTGCTGCGGACTGAT
	ISlllla-P	5-FAM-CGTGTGGAGGAGCGAACCATTGGTAT-TAMRA
*S. abortus equi*	Fljb-F	CATTAGGCAACCCGACAGTA
	Fljb-R	GGTAGCACCGAATGATACAGC
	Fljb-P	6-FAM-ACTGTAAGTGGTTATACCGATGC-MGB
*Brucella* spp.	bcsp31-F	GCTCGGTTGCCAATATCAATGC
	bcsp31-R	GGGTAAAGCGTCGCCAGAAG
	bcsp31-P	6-FAM-AAATCTTCCACCTTGCCCTTGCCATCA-BHQ1

**Table A3:** Real-time PCR reaction procedure.

Target Pathogen	Reaction Volume (μL)	Mix Components (per Reaction)	Thermal Program
*Chlamydia* spp.	20	Upstream primer 0.4 μL; Downstream primer 0.4 μL; Probe 0.2 μL; Enzyme 10 μL; DNA template 5 μL; ddH_2_O to 20 μL	Pre-denaturation: 95 °C, 5 min; PCR cycling (×45): 95 °C 10 s, 60 °C 30 s
*C. burnetii*	20	Upstream primer 0.4 μL; Downstream primer 0.4 μL; Probe 0.2 μL; Enzyme 10 μL; DNA template 5 μL; ddH_2_O to 20 μL	Pre-denaturation: 95 °C, 5 min; PCR cycling (×45): 95 °C 10 s, 60 °C 30 s
*S. abortus equi*	20	Upstream primer 0.4 μL; Downstream primer 0.4 μL; Probe 0.2 μL; Enzyme 10 μL; DNA template 5 μL; ddH_2_O to 20 μL	Pre-denaturation: 95 °C, 5 min; PCR cycling (×45): 95 °C 10 s, 60 °C 30 s
*Brucella* spp.	20	Upstream primer 0.4 μL; Downstream primer 0.4 μL; Probe 0.2 μL; Enzyme 10 μL; DNA template 5 μL; ddH_2_O to 20 μL	Pre-denaturation: 95 °C, 5 min; PCR cycling (×45): 95 °C 10 s, 60 °C 30 s

**Table A4:** Antibiotic resistance genes correspond to antibiotics.

Antibiotic	Sulfonamides	Trimethoprim	Fluoroquinolones	ESBL
Genes	*sul1*, *sul2*	*dfrA1*, *dfrA5*	*qnrA*, *qnrB*	*SHV*, *CTX-M-1/2/9/8*
Antibiotic	Penicillin	Carbapenems	Macrolide/Lincosamide/Streptogramin	Tetracyclines
Genes	*TEM-1*, *OXA-1*	*IMP-1/2*, *NDM-1*, *VIM-1/2*, *OXA-23/48/58*, *KPC-3*	*ermA/B/C*	*TetM*
Antibiotic	Vancomycin	Methicillin/Penicillin		
Genes	*vanA/B/C*	*mecA/B/C*		
